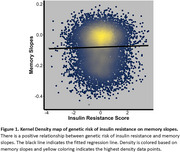# Genetic associations between insulin resistance and memory performance/decline: A NACC cohort study

**DOI:** 10.1002/alz70860_106817

**Published:** 2025-12-23

**Authors:** Emma Nolan, Ashley Sawyer, Derek B. Archer, Logan Dumitrescu, Walter W. Kukull, Sarah Biber, Paul K Crane, Shubhabrata Mukherjee, Jesse Mez, Seo‐Eun Choi, Brandon Klinedinst, Michael L. Lee, Phoebe Scollard, Elizabeth C. Mormino, Sterling C Johnson, Timothy J. Hohman

**Affiliations:** ^1^ Vanderbilt Memory and Alzheimer's Center, Vanderbilt University Medical Center, Nashville, TN, USA; ^2^ Epidemiology Doctoral Program, School of Medicine, Vanderbilt University, Nashville, TN, USA; ^3^ Vanderbilt Genetics Institute, Vanderbilt University Medical Center, Nashville, TN, USA; ^4^ Department of Neurology, Vanderbilt University Medical Center, Nashville, TN, USA; ^5^ Department of Epidemiology, University of Washington School of Public Health, Seattle, WA, USA; ^6^ University of Washington, Seattle, WA, USA; ^7^ National Alzheimer's Coordinating Center, University of Washington, Seattle, WA, USA; ^8^ Department of Medicine, University of Washington, Seattle, WA, USA; ^9^ Department of Neurology, Boston University Chobanian & Avedisian School of Medicin, Boston, MA, USA; ^10^ Alzheimer's Disease Research Center, Boston University Chobanian & Avedisian School of Medicine, Boston, MA, USA, Boston, MA, USA; ^11^ Department of Medicine, University of Washington School of Medicine, Seattle, WA, USA; ^12^ Department of Neurology and Neurological Sciences, Stanford University, Stanford, CA, USA; ^13^ Wisconsin Alzheimer's Institute, University of Wisconsin School of Medicine and Public Health, Madison, WI, USA; ^14^ Department of Pharmacology, Vanderbilt University School of Medicine, Nashville, TN, USA

## Abstract

**Background:**

Insulin resistance (IR), characterized by reduced insulin responsiveness, has been hypothesized to be related with subsequent cognitive decline beyond the pace of normal aging, increasing the risk for all‐cause dementia. Polygenic risk scores (PRS) can provide a genetic risk profile for IR. This study aimed to cross‐sectionally/longitudinally relate IR genetic liability to memory performance.

**Method:**

Using summary statistics from a published genome‐wide association study (*Oliveri et al*., 2024; *N* = 402,398, UK Biobank) of the triglyceride to high‐density lipoprotein cholesterol ratio (TG:HDL‐C; an indicator of IR), a PRS was built in 22,572 participants from the National Alzheimer's Coordinating Center (NACC) cohort (8,456 variants included with a *p*‐value threshold of ≤0.001). Linear regression evaluated baseline memory while linear mixed‐effect models assessed baseline interactions with longitudinal memory decline. Models were adjusted for sex, baseline age, Body Mass Index (BMI) (kg/m^2^), and *APOE*‐ɛ4 positivity. Restricted cubic splines and scaling accounted for non‐linearity in age and BMI. Missingness in BMI and *APOE*‐ɛ4 positivity was addressed via multiple imputation. Sensitivity analyses excluded individuals with AD‐related comorbidities (e.g., stroke, cancer, Parkinson's disease, traumatic brain injury, etc.) (*N* = 8,227) and stratified by *APOE*‐ɛ4 and *APOE*‐ε2 positivity.

**Result:**

A cohort of 10,806 non‐Hispanic White adults (median age 73.5), had mean BMI of 26.7; 38.4% were *APOE*‐ɛ4 carriers, 52.7% were female, and 37.4% were cognitively impaired at baseline. A positive association was observed between genetic risk for TG:HDL‐C and baseline memory (β=0.019, *p* = 0.009). Additionally, a higher TG:HDL‐C PRS was associated with slower memory decline (β=0.003, *p* = 0.040; Figure 1). In sensitivity models, removing participants with comorbidities attenuated associations by 26%, driven primarily by cancer or traumatic head injury. Stratified analyses revealed stronger associations for *APOE*‐ɛ4 and ɛ2 non‐carriers than carriers for both baseline and longitudinal memory.

**Conclusion:**

Genetic liability to IR as indicated by a TG:HDL‐C PRS is associated with a small, but significant, association with better memory performance and slower memory decline. However, this effect is modified by comorbidities and *APOE*‐ɛ4 and ɛ2 non‐carrier status, suggesting a complex interplay between genetic risk for IR, comorbidities, as well as triglyceride and cholesterol metabolism. Future work with larger populations may clarify these relationships